# Individualized survival predictions using state space model with longitudinal and survival data

**DOI:** 10.1098/rsif.2023.0682

**Published:** 2024-07-31

**Authors:** Mark Cauchi, Andrew R. Mills, Allan Lawrie, David G. Kiely, Visakan Kadirkamanathan

**Affiliations:** ^1^ Department of Automatic Control and Systems Engineering, The University of Sheffield, Mappin Street, Sheffield S1 3JD, UK; ^2^ National Heart and Lung Institute, Imperial College London, Dovehouse Street, London SW3 6LY, UK; ^3^ Sheffield Pulmonary Vascular Disease Unit, Royal Hallamshire Hospital Sheffield, NIHR Biomedical Research Centre Sheffield and Department of Clinical Medicine, The University of Sheffield, Beech Hill Road, Sheffield S10 2RX, UK

**Keywords:** expectation maximization algorithm, joint model, longitudinal data, pulmonary arterial hypertension, state space model, survival data

## Abstract

Monitoring disease progression often involves tracking biomarker measurements over time. Joint models (JMs) for longitudinal and survival data provide a framework to explore the relationship between time-varying biomarkers and patients’ event outcomes, offering the potential for personalized survival predictions. In this article, we introduce the linear state space dynamic survival model for handling longitudinal and survival data. This model enhances the traditional linear Gaussian state space model by including survival data. It differs from the conventional JMs by offering an alternative interpretation via differential or difference equations, eliminating the need for creating a design matrix. To showcase the model’s effectiveness, we conduct a simulation case study, emphasizing its performance under conditions of limited observed measurements. We also apply the proposed model to a dataset of pulmonary arterial hypertension patients, demonstrating its potential for enhanced survival predictions when compared with conventional risk scores.

## 1. Introduction

Safeguarding and enhancing patients’ well-being is of paramount importance in healthcare, often necessitating ongoing monitoring. In recent years, numerous medical institutions have opted to store patients’ information in electronic health records (EHRs) databases [1,2]. As time progressed, the potential inherent within this wealth of raw data, encompassing data from thousands of patients, became increasingly evident to researchers. These databases encompass a wide range of information, including diagnostic tests, laboratory results, medical procedures, their respective outcomes, and other medical occurrences that patients may encounter over their lifetimes [2–4]. The availability of such extensive healthcare data is progressively turning the aspiration of personalized treatment into a tangible reality.

Despite its advantages, EHR modelling is still relatively simplistic due to the difficulty encountered in applying conventional statistical methods [5,6]. Obtaining an accurate, parsimonious and explanatory model is hindered by many characteristics of EHRs, including heterogeneity, irregular timing of events, missing data, and lack of standardization, among others [1,2,4–6]. That being said, these databases typically contain richer and more realistic symptoms dynamics, additional biomarkers, and more frequent visits, when compared to clinical trials [1]. Despite the challenges posed by modelling such complex data, successful achievement in this endeavour holds the promise of enabling more precise outcome predictions and, consequently, the realization of personalized medicine.

Disease monitoring often involves tracking specific biomarkers that medical experts have identified as significant indicators, drawing on their experience to gauge a disease’s progression [7]. In contemporary healthcare practices, these biomarker values are commonly employed in constructing risk scores, with the resultant score indicating the patient’s present disease stage [8,9]. However, this approach typically overlooks the underlying dynamics and trajectory of biomarkers. When dynamics are considered, they are usually simplified, such as assessing changes in biomarker values over a 1-year period [10].

The state space model (SSM) serves as a versatile tool for capturing the temporal evolution and measurement processes within a probabilistic framework. It consists of two key components: a dynamics equation, which describes how hidden states evolve over time, and an observation equation, illustrating the connection between these continuous-valued hidden states and the observed biomarkers. The strength of the SSM lies in its ability to employ first-order difference equations, making it computationally efficient and appealing [11,12]. Consequently, it has found success in various domains, including engineering [13], life sciences [14], social sciences [15], econometrics [16] and healthcare [17]. Interestingly, its efficacy has extended to movement ecology as well, where SSMs have gained more popularity than linear mixed effects (LME) models, becoming the preferred choice [18,19]. In the realm of healthcare, the SSM has been employed for diverse purposes, including the reconstruction of EEG and MEG signals [20,21], decoding ensemble spikes in neuroscience [22,23], classification and prediction of clinical data [24], and general clinical monitoring [25], among other applications. Notably, SSMs have primarily been applied to relatively short-term observational data, and their use in modelling irregular time-series spanning months or years has been limited.

A common healthcare application involves time-dependent biomarkers for monitoring diseases, such as cluster of differentiation 4 (CD4) counts for human immunodeficiency virus [26–28], prostate-specific antigen for predicting prostate cancer recurrence risk [29–31], and echocardiogram variables for assessing cardiovascular diseases [32–34]. Early attempts to model survival in these scenarios often involved directly integrating these time-varying measurements into the time-dependent Cox proportional hazards model. However, it became evident that such an approach introduced bias [35,36]. Subsequently, researchers turned to a two-stage approach, initially modelling the longitudinal process and then integrating the output into a survival model [37]. Unfortunately, this did not eliminate bias, prompting further exploration of alternatives [26,38,39].

The joint model (JM) for longitudinal and survival data emerged as a solution to this bias issue [26]. JM consists of two sub-models: the longitudinal process, often represented by a mixed effects model, and the survival process, typically modelled using the Cox proportional hazards model. These two processes are linked through random effects, with the key assumption that, given these random effects, the longitudinal and survival processes are conditionally independent [26]. These random effects are personalized for each patient, allowing deviations from the population trajectory. JM has been successfully applied in various disease monitoring contexts, including those involving the biomarkers mentioned earlier.

In this study, we extend the SSM to incorporate survival data for patient health modelling and survivability assessment. We enhance the canonical SSM [40] by introducing a proportional hazards model influenced by the hidden states. Drawing inspiration from the JM expectation maximization (EM) algorithm [26], we present the linear state space dynamic survival model (LSDSM) with Gauss–Markovian assumptions. LSDSM diverges from JM mainly in its representation of longitudinal biomarkers as a dynamic model, as opposed to relying on basis functions. Furthermore, LSDSM provides coefficients associated with previous hidden state values, in contrast to the time-dependent covariates employed in the LME model.

The advantages of SSMs are manifold: (i) they serve as generalizations of various time-series models like autoregressive (AR) and autoregressive integrated moving average (ARIMA) models; (ii) they can model time series without necessitating covariates or design matrices, although they can be included seamlessly; (iii) they can accommodate expert knowledge by allowing the fixation of sub-structures; (iv) they maintain interpretability with appropriate matrix selection; and (v) they offer flexibility in altering temporal order without changing the estimation procedure [41,42]. Furthermore, SSMs inherently distinguish between process variation and measurement error, aiding in the identification of true underlying processes [19]. They also naturally account for correlation structures between measurements and sequential time points, alleviating the need for precise pre-specification of such correlations, as is often required in LME models [12]. Using an SSM presents certain drawbacks when compared with LME models. Specifically, in scenarios where longitudinal observations are sparse, basis functions offer a natural interpolation capability, while the state space approach necessitates handling missing data. Additionally, the linear state space framework is constrained by specific longitudinal patterns, although its smoothing capabilities may alleviate this limitation when dealing with sparse data. However, it is worth noting that for certain applications, these drawbacks are relatively minor and can be addressed effectively, such as incorporating smoothness priors and/or extending to nonlinear SSMs. Moreover, wearable technologies are gaining prominence in healthcare, enabling the collection of more frequent and regular longitudinal time-series data, a domain in which SSMs excel in effectively modelling such information. Finally, it is worth noting that SSMs focus on the connection between the current and future time steps, simplifying the forecasting of future values even beyond the observation period [16]. In this paper, we harness these advantages, coupled with the computational efficiency of SSMs, to formulate an estimation framework and investigate the performance of LSDSM through simulations and a real-world application.

The remainder of the paper is structured as follows. Section 2 provides an overview of the notation and methodology underpinning the LSDSM framework. In §3, we delve into the estimation process and lay out the critical assumptions made. Performance metrics for assessing the predictive capabilities of the models, along with the procedure for individualized survival predictions, are detailed in §4. Moving on to §5, we present the results of our simulation studies. In §6, we pivot our focus to an application involving patients with pulmonary arterial hypertension (PAH), where we discuss the analysis conducted using LSDSM and compare its performance against the established risk score approach. The paper concludes with a summary of findings and a broader discussion in the final section.

## 2. Linear state space dynamic survival model

The linear state space dynamic survival model is constructed using two sub-processes, these being the longitudinal and survival processes. The aim of the former process is to reveal the true biomarker values in the presence of measurement error, while the objective of the survival process is to identify the relationship of the true biomarker values and other covariates of interest, with the hazard of the patient. For ease of reference, the notation is listed in table 4 in appendix A.

In this work, we use a discrete-time SSM for the longitudinal sub-process, while also introducing the survival sub-process to the model through the form of a proportional hazards model. Thus, we are proposing a Markov-based dynamic model for the longitudinal process that has the potential to capture the rate of variations. In other words, the current true biomarker values will be a function of previous biomarker values, as an alternative to the design matrices that involve basis functions of time in the JM.

The hidden states trajectories $x_{i,j}\in R^{m_{x}\times 1}$ include the true underlying biomarkers, and they are dictated by the Markovian assumption, i.e. that the current state values are a function of the state values at the previous time point. In this work, we shall make use of the linear Gaussian SSM with the following dynamic and observation equations:

2.1 $$\begin{array}{cc} x_{i,j+1}\; & =Ax_{i,j}+w_{i,j}\\ \mathrm{and}\;\;\;\;y_{i,j} & =Cx_{i,j}+v_{i,j} \end{array}\}\;\;\;\;$$

where *i* and *j* represent the *i*th patient and the *j*th time step, respectively, $A\in R^{m_{x}\times m_{x}}$ is the transition matrix dictating the dynamics of the longitudinal hidden states in time, and $w_{i,j}\in R^{m_{x}\times 1}$ is the disturbance term vector that allows variation from the population trajectory. $y_{i,j}\in R^{m_{y}\times 1}$ is the observation vector containing a list of measurements for patient *i* at time step *j*, $C\in R^{m_{y}\times m_{x}}$ is referred to as the observation matrix which provides a relationship between the observation and hidden state vectors, while $v_{i,j}\in R^{m_{y}\times 1}$ is the measurement error vector, sampled from a zero-mean Gaussian distribution N(0,V).

Note that in the first expression of equation (2.1), $x_{i,j}$ and $x_{i,j+1}$ are separated by a constant time step Δ*t*. The number of hidden states may exceed the number of available biomarkers, offering the potential to capture higher-order dynamics. In our model, we characterize the true biomarker trajectory using an AR(*M*) process where *M* is the AR order, resulting in a total of *m*_*x*_ = *M* × *m*_*y*_ states. Consequently, we structure $C=[I\;0]\in R^{m_{y}\times m_{x}}$, where $I\in R^{m_{y}\times m_{y}}$ represents an identity matrix. This construction allows for a direct association between the hidden states and the observed biomarkers. It is important to note that C remains fixed, and therefore is not included in the set of parameters to be estimated. Complementing this structure in C, the A matrix is chosen to maintain the SSM in a canonical form, ensuring identifiability and obtaining a unique solution [40]:

2.2 $$A=\left[\begin{array}{rl} & \;\;\bar{A}\\ & I\;0 \end{array}\right]$$

This special structure adopts simple AR behaviour. As an example, for an AR(2) process, *m*_*x*_ = 2 × *m*_*y*_ where *m*_*y*_ is the number of unique biomarkers observed. Thus, for this A matrix, $\bar{A}\in R^{m_{y}\times m_{x}}$, $I\in R^{(M-1)m_{y}\times (M-1)m_{y}}$, and $0\in R^{(M-1)m_{y}\times m_{y}}$.

Note that an additional error term is introduced in the dynamics equation, $w_{i,j}$, which is typically referred to as the disturbance or uncertainty term, and it captures the deviations represented by the dynamics A, which in turn is determined from the whole population. This is assumed to be sampled from a zero-mean normal distribution N(0,W). This random walk effect allows patients’ trajectories to deviate from the population, and thus provides individualized longitudinal components for every patient. In the canonical representation specified above, this disturbance is assumed to influence solely the first *m*_*y*_ states. This leads to $W=G\overset{˘}{W}G^{⊤}$ where $G={\left[I\;0\right]}^{⊤}\in R^{m_{x}\times m_{y}}$, incorporating an *m*_*y*_ × *m*_*y*_ identity matrix, I. Here, $\overset{˘}{W}$ denotes the reduced disturbance variance that impacts the first *m*_*y*_ hidden states, while the trailing hidden states are assumed deterministic.

The survival model used for LSDSM is the proportional hazards model. The typical approach to include longitudinal information is to take the current value of the true biomarker trajectory, but many different associations may be implemented. For an enhanced list of these associations, the reader is referred to the review by Hickey *et al.* [43]. For this model, the hidden states representing the true underlying biomarker trajectories are used for the current hazard calculation. Hence, the survival sub-process is governed by the following hazard function:

2.3 $$h_{i}(t_{i,j})=\exp\left\{\gamma^{⊤}\omega_{i}+\alpha^{⊤}Hx_{i,j}\right\},$$

where *h*_*i*_ (*t*) is the hazard value for patient *i* at time *t*, $\omega_{i}\in R^{m_{\omega }\times 1}$ is the set of baseline covariates for patient *i*, and $\gamma \in R^{m_{\omega }\times 1}$ and $\alpha \in R^{m_{\alpha }\times 1}$ are the coefficients linking the baseline covariates and the hidden states to the hazard function, respectively.

The matrix $H\in R^{m_{\alpha }\times m_{x}}$ permits a linear combination of the hidden states to influence the hazard function. This flexibility in the model structure allows for the incorporation of specific hidden states in modulating the hazard function. Additionally, the introduction of changes in the true biomarker value as potential time-dependent covariates within the hidden states can be achieved using the matrix H. The determination of H is undertaken by the analyst, who relies on their expert insights to identify which associations are likely to exert an influence on the patient’s probability of survival. The survival function is related to the hazard function as

2.4 $$S_{i}(t_{i,j})=\exp\left\{-\int_{0}^{t_{i,j}}h_{i}(s)\;ds\right\}.$$

This formulation allows for an individualization of the survival curves as a function of the baseline covariates ($\omega_{i}$) and the true biomarkers trajectories ($x_{i,j}$) for patient *i*.

The assumptions represented by this model and additional assumptions required to facilitate the estimation procedure are:

**A.1** Markovian assumption for the longitudinal sub-process;
**A.2** conditional independence such that given the hidden state values, the longitudinal and survival processes are independent;
**A.3** observation times and censoring are not affected by the hidden states values (i.e. they are observed or missing at random); and
**A.4** the hidden state values remain constant between time steps, i.e. x(jΔt)=x((j+1)Δt−ϵ), where ϵ is some small number ≪ Δ*t*, leading to a simplification in the estimation procedure.

## 3. Estimation procedure

In our effort to integrate and monitor survival data within the extended SSM, we build upon the foundational work introduced by Dewar & Kadirkamanathan [40]. We also derive inspiration from the EM algorithm, a well-established maximum-likelihood approach repeatedly employed in JMs [26]. Consequently, we further develop the EM algorithm tailored for the SSM by seamlessly incorporating survival data as an additional set of accessible observations.

### 3.1. Expectation maximization algorithm

Our proposed model employs a discrete-time SSM to monitor the true underlying biomarkers. Here, the current states serve as a representation for predicting the patient’s future health outcome. This framework introduces novel probability distributions within the observed likelihood expression. Similar to the conditional independence assumption commonly used in JMs, we maintain the independence between longitudinal and survival data given the values of the hidden states. Operating within the maximum-likelihood paradigm, our objective is to maximize the likelihood of the observed data:

3.1 $$L(\theta )=p(D^{\left(o\right)};\theta )=\prod_{i=1}^{n}p(D_{i}^{\left(o\right)};\theta ).$$

The above equation assumes that all patient data are independent of each other, and hence we can simply multiply their likelihoods. On the basis of assumption **A.3**, the hospital visiting process and censoring are assumed non-informative [38,44] and hence may be disregarded. Equation (3.1) simplifies to

3.2 $$L(\theta )=\prod_{i=1}^{n}p(Y_{i},T_{i},\delta_{i};\theta ).$$

Using assumption **A.2**, the likelihood can be expressed as

3.3 $$L(\theta )=\prod_{i=1}^{n}\int_{-\infty }^{\infty }p(Y_{i}|X_{i};\theta )p(T_{i},\delta_{i}|X_{i};\theta )p(X_{i};\theta )\;dX_{i}$$

and the log likelihood, which is typically easier to maximize, is given by

3.4 $$l(\theta )=\log\{L(\theta )\}=\sum_{i=1}^{n}\log\left\{\int_{-\infty }^{\infty }p(Y_{i}|X_{i};\theta )p(T_{i},\delta_{i}|X_{i};\theta )p(X_{i};\theta )\;dX_{i}\right\}.$$

**Figure 1.**
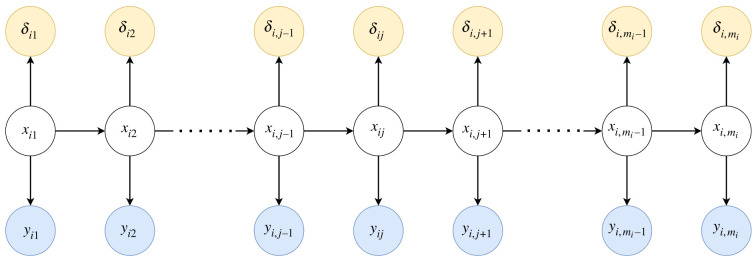
Graphical model for the state space model for longitudinal and survival data representing the causal relations influencing the latent complete patient trajectories. White circles indicate latent variables, while shaded circles are observed variables.

Using assumption **A.4**, the graphical model of the proposed framework is simplified as shown in figure 1, where ${\bar{\delta }}_{i,j}=I(t_{i,j}<T_{i}\leq t_{i,j+1},\delta_{i}=1)$, and *I*( · ) is equal to 1 if all conditions inside the brackets are satisfied, and zero otherwise, and $x_{i,j}$ and $y_{i,j}$ are the hidden states and observed biomarkers vectors for patient *i* at time step *j*, respectively. Assuming a linear Gaussian SSM and a proportional hazards survival model for the longitudinal and survival processes, respectively, the conditional probabilities used for this framework are defined as follows:

3.5 $$\begin{array}{rl} \;p(T_{i},\delta_{i}|X_{i};\theta ) & =h{\left(T_{i}\right)}^{\delta_{i}}S(T_{i})\\ & =\prod_{\;j=1}^{m_{i}}p(t_{i,j+1},{\bar{\delta }}_{i,j}|x_{i,j})\\ & =\prod_{\;j=1}^{m_{i}}\exp\{{\bar{\delta }}_{i,j}\gamma^{⊤}\omega_{i}+{\bar{\delta }}_{i,j}\alpha^{⊤}Hx_{i,j}-\tau_{i,j}\exp\left\{\gamma^{⊤}\omega_{i}+\alpha^{⊤}Hx_{i,j}\right\}\}, \end{array}$$

3.6 $$\begin{array}{rl} \;p(Y_{i}|X_{i};\theta ) & =\prod_{\;j=1}^{m_{i}}p(y_{i,j}|x_{i,j})\\ & =\prod_{\;j=1}^{m_{i}}{\left(2\pi \right)}^{-m_{y}/2}|V{|}^{-1/2}\times \exp\left\{-\frac{1}{2}{\left(y_{i,j}-Cx_{i,j}\right)}^{⊤}V^{-1}(y_{i,j}-Cx_{i,j})\right\} \end{array}$$

3.7 $$\begin{array}{rl} \mathrm{and}\;\;\;p(X_{i};\theta ) & =p(x_{i1})\prod_{\;j=2}^{m_{i}}p(x_{i,j}|x_{i,j-1})\\ & ={\left(2\pi \right)}^{-m_{x}/2}|{\bar{W}}_{1}{|}^{-1/2}\exp\left\{-\frac{1}{2}{\left(x_{i1}-{\bar{x}}_{1}\right)}^{⊤}{\bar{W}}_{1}^{-1}(x_{i1}-{\bar{x}}_{1})\right\}\\ & \;\times \prod_{\;j=2}^{m_{i}}{\left(2\pi \right)}^{-m_{x}/2}|W{|}^{-1/2}\times \exp\left\{-\frac{1}{2}{\left(x_{i,j}-Ax_{i,j-1}\right)}^{⊤}W^{-1}(x_{i,j}-Ax_{i,j-1})\right\}, \end{array}$$

where $X_{i}$ and $Y_{i}$ are the sets of all values of the patient’s true and observed biomarkers, respectively. Equation (3.7) shows the distributions for the general SSM. In using the outlined canonical representation of SSM, this would require slight modifications, as shown in §1 of the electronic supplementary material, where we would require the probability distribution of the leading *m*_*y*_ hidden values, labelled as $X_{i}^{*}$. Note that in using a discrete-time SSM, the biomarker measurements are structured to follow a regular time series with Δ*t* as the pseudo-sampling time, representing the chosen discrete-time points at which the true biomarkers are estimated. If the biomarker measurements are captured at irregular time intervals, these can be binned as observations at the chosen time steps. If no observations are made in a time interval, they are treated as missing measurements. Hence for every patient, we observe *m*_*i*_ = ceil(*T*_*i*_/Δ*t*) measurements, some are typically missing.

The decomposition of equation (3.5) is made possible using assumption **A.4**, where the components of the survival probability distribution can be defined as shown in equations (3.8) and (3.9):

3.8 $$\begin{array}{rl} h{\left(T_{i}\right)}^{\delta_{i}} & =\exp{\left\{\gamma^{⊤}\omega_{i}+\alpha^{⊤}x_{i}(T_{i})\right\}}^{\delta_{i}}\\ & =\prod_{\;j=1}^{m_{i}}\exp{\left\{\gamma^{⊤}\omega_{i}+\alpha^{⊤}x_{i,j}\right\}}^{{\bar{\delta }}_{i,j}}\\ & =\prod_{\;j=1}^{m_{i}}\exp\left\{{\bar{\delta }}_{i,j}\gamma^{⊤}\omega_{i}+{\bar{\delta }}_{i,j}\alpha^{⊤}Hx_{i,j}\right\} \end{array}$$

and

3.9 $$\begin{array}{rl} S(T_{i}) & =\exp\left\{-\int_{0}^{T_{i}}\exp\left\{\gamma^{⊤}\omega_{i}+\alpha^{⊤}Hx_{i}(\tau )\right\}\;d\tau \right\}\\ & =\exp\left\{-\sum_{\;j=1}^{m_{i}}\tau_{i,j}\exp\left\{\gamma^{⊤}\omega_{i}+\alpha^{⊤}Hx_{i,j}\right\}\right\}\\ & =\prod_{\;j=1}^{m_{i}}\exp\left\{-\tau_{i,j}\exp\left\{\gamma^{⊤}\omega_{i}+\alpha^{⊤}Hx_{i,j}\right\}\right\}. \end{array}$$

**Figure 2.**
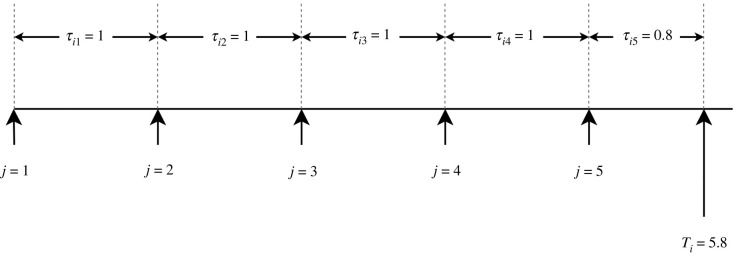
An example showcasing how *τ*_*i*,*j*_ is calculated for every observation period.

A patient may have a maximum of one ${\bar{\delta }}_{i,j}$ equal to 1, and it can be easily verified that the third line in equation (3.8) is equivalent to the first. Note that *T*_*i*_ may be located between SSM time steps, and thus *τ*_*i*,*j*_ = Δ*t* for all periods except the last one, where $\tau_{i,m_{i}}=T_{i}-m_{i}\Delta t$. An example diagram is shown in figure 2. Also, note that even though the time step for the last value is not regular, for convenience the final recorded time for patient *i* is denoted as $t_{i,m_{i}+1}=T_{i}$.

This decomposition of the survival probability distribution can be explained as follows. If the survival data are split according to the respective time steps, then every element within the product of equation (3.5) is identifying the distribution for the patient surviving or experiencing the event within that time frame. Hence, it can be expressed as the probability distribution including only survival data within the next time step, i.e. $p(t_{i,j+1},{\bar{\delta }}_{i,j}|x_{i,j})$. Note that this is a function of only the values of the current hidden states $x_{i,j}$. This decomposition is advantageous since in using the inherent properties of the SSM, the current observation also depends solely on the current hidden states values [11]. This leads to a simplification in the joint probability distribution.

**Expectation step:** The proposed observed data log likelihood is expressed as

3.10 $$l(\theta )=\log\{L(\theta )\}=\sum_{i=1}^{n}\log\left\{\int_{-\infty }^{\infty }p(Y_{i}|X_{i};\theta )p(T_{i},\delta_{i}|X_{i};\theta )p(X_{i};\theta )\;dX_{i}\right\}.$$

This is computationally difficult to maximize directly with respect to the parameters, due to the integration being located inside the log function [45]. Thus, we resort to the EM algorithm. In the EM algorithm, the goal is to maximize a lower bound to the observed data log likelihood. This requires the expectation of the complete data log likelihood with respect to the unobserved parts of the complete dataset. Since some time steps may contain no biomarker measurements, then some $y_{i,j}$ may be unobserved, and should be treated as ‘missing’. Let $Y_{i}^{\left(O\right)}$ represent the set of observed measurements, while $Y_{i}^{\left(M\right)}$ denote the set of missing measurements for patient *i* [11,46]. Then the required expectation is with respect to the distribution $p(X_{i},Y_{i}^{\left(M\right)}|Y_{i}^{\left(O\right)},T_{i},\delta_{i};\theta^{\left(k\right)})$ for every patient, where *k* is the current iteration in the EM algorithm. From now on, we shall use the shorthand notation *E*[ · ] to represent $E_{X_{i},Y_{i}^{\left(M\right)}|Y_{i}^{\left(O\right)},T_{i},\delta_{i};\theta^{\left(k\right)}}[\cdot ]$, unless stated otherwise. Also, all probability distributions are with respect to the unknown values of the parameter values, and thus p( ⋅ ;θ) shall be expressed as *p*( · ) unless stated otherwise. Hence, the expectation of the complete data log likelihood can be expressed as

3.11 $$E[l(\theta |D^{\left(c\right)})]=\sum_{i=1}^{n}E[\log\{p(T_{i},\delta_{i}|X_{i})\}]+E[\log\{p(Y_{i}|X_{i})\}]+E[\log\{p(X_{i})\}].$$

Using the distributions defined in equations (3.5)–(3.7), the above expectation simplifies to (ignoring additive constants)

3.12 $$\begin{array}{rl} E[l(\theta |D^{\left(c\right)},X)] & =\sum_{i=1}^{n}\left(\sum_{\;j=1}^{m_{i}}({\bar{\delta }}_{i,j}\gamma^{⊤}w_{i}+{\bar{\delta }}_{i,j}\alpha^{⊤}H\;E[x_{i,j}]-\tau_{i,j}\exp\left\{\gamma^{⊤}w_{i}\right\}E[\exp\left\{\alpha^{⊤}Hx_{i,j}\right\}])\right)\\ & \;-\frac{1}{2}\sum_{i=1}^{n}\left(\sum_{\;j=1}^{m_{i}}(\log|V|+E[{\left(y_{i,j}-Cx_{i,j}\right)}^{⊤}V^{-1}(y_{i,j}-Cx_{i,j})])\right)\\ & \;-\frac{1}{2}\sum_{i=1}^{n}((\log|{\bar{W}}_{1}|+E[{\left(x_{i1}-{\bar{x}}_{1}\right)}^{⊤}{\bar{W}}_{1}^{-1}(x_{i1}-{\bar{x}}_{1})]))\\ & \;-\frac{1}{2}\sum_{i=1}^{n}\left(\sum_{\;j=2}^{m_{i}}(\log|W|+E[{\left(x_{i,j}-Ax_{i,j-1}\right)}^{⊤}W^{-1}(x_{i,j}-Ax_{i,j-1})])\right). \end{array}$$

Maximizing the expectation of the complete data log likelihood with respect to the parameters requires the evaluation of the following expectations:

3.13 $$E[x_{i,j}]={\hat{\mu }}_{i,j}$$

3.14 $$E[x_{i,j}x_{i,j}^{⊤}]={\hat{\Sigma }}_{i,j}+{\hat{\mu }}_{i,j}{\hat{\mu }}_{i,j}^{⊤}$$

3.15 $$E[x_{i,j}x_{i,j-1}^{⊤}]={\hat{\Sigma }}_{i,j}J_{i,j-1}^{⊤}+{\hat{\mu }}_{i,j}{\hat{\mu }}_{i,j-1}^{⊤}$$

3.16 $$E[x_{i,j}^{*}x_{i,j}^{*⊤}]={\hat{\Sigma }}_{i,j}^{*}+{\hat{\mu }}_{i,j}^{*}{\hat{\mu }}_{i,j}^{*⊤}$$

3.17 $$E[x_{i,j}^{*}x_{i,j-1}^{⊤}]=M_{i,j}^{*}+{\hat{\mu }}_{i,j}^{*}{\hat{\mu }}_{i,j-1}^{⊤}$$

3.18 $$E[\exp\left\{\alpha^{⊤}Hx_{i,j}\right\}]\approx \exp\left\{\alpha^{⊤}H{\hat{\mu }}_{i,j}+\frac{1}{2}\alpha^{⊤}H{\hat{\Sigma }}_{i,j}H^{⊤}\alpha \right\}$$

3.19 $$E[y_{i,j}]=y_{i,j}-\nabla_{i,j}(y_{i,j}-CE[x_{i,j}])$$

3.20 $$E[y_{i,j}y_{i,j}^{⊤}]=I_{i,j}^{\left(M\right)}(\nabla_{i,j}V+\nabla_{i,j}C{\hat{\Sigma }}_{i,j}C^{⊤}\nabla_{i,j}^{⊤})I_{i,j}^{\left(M\right)}+E[y_{i,j}]E{\left[y_{i,j}\right]}^{⊤}$$

3.21 $$\mathrm{and}\;\;\;\;\;\;\;\;\;E[y_{i,j}x_{i,j}^{⊤}]=\nabla_{i,j}C{\hat{\Sigma }}_{i,j}+E[y_{i,j}]E{\left[x_{i,j}\right]}^{⊤},$$

where ${\hat{\mu }}_{i,j}$ and ${\hat{\Sigma }}_{i,j}$ are the expected mean and variance of the hidden state values, $M_{i,j}={\hat{\Sigma }}_{i,j}J_{i,j-1}^{⊤}$, and * here refers to the reduced vectors and matrices, retaining information only about the first *m*_*y*_ states. These are used for the parameter updates of the canonical representation of SSM. $I_{i,j}^{\left(M\right)}$ is the identity matrix with zeros corresponding to the rows and columns of observed measurements for time step *j*, $\nabla_{i,j}=I-V{\left(\Omega_{i,j}^{\left(O\right)}\right)}^{⊤}{\left(V^{\left(O,O\right)}\right)}^{-1}\Omega_{i,j}^{\left(O\right)}$, having $\Omega_{i,j}^{\left(O\right)}$ be a matrix extracting only the observed parts of the $y_{i,j}$ vector, and $V^{\left(O,O\right)}$ be the biomarker measurement error variance retaining the rows and columns corresponding to the observed parts of the vector $y_{i,j}$ [46].

The first five expectations in the list could have been derived using Rauch–Tung–Striebel (RTS) smoother, had the observations been only longitudinal [47,48]. The presence of survival data demands the introduction of a suitable estimator to compute these expectations. Using Bayes’ theorem, the posterior distribution of the hidden state at time *j* given the observed data up to that time can be expressed as

3.22 $$\begin{array}{rl} & p(x_{i,j}|y_{i,1\;:\;j},t_{i,j+1},{\bar{\delta }}_{i,1\;:\;j})=p(x_{i,j}|y_{i,j},t_{i,j+1},\delta_{i,j},(y_{i,1\;:\;j-1},t_{i,j},{\bar{\delta }}_{i,1\;:\;j-1}))\\ & \;=\frac{\;p(y_{i,j},t_{i,j+1},{\bar{\delta }}_{i,j}|x_{i,j},(y_{i,1\;:\;j-1},t_{i,j},{\bar{\delta }}_{i,1\;:\;j-1}))p(x_{i,j}|(y_{i,1\;:\;j-1},t_{i,j},{\bar{\delta }}_{i,1\;:\;j-1}))}{p(y_{i,j},t_{i,j+1},{\bar{\delta }}_{i,j}|(y_{i,1\;:\;j-1},t_{i,j},{\bar{\delta }}_{i,1\;:\;j-1}))}. \end{array}$$

The probability distribution in the denominator may be ignored as it is only dependent on the data and acts as a normalization constant. Hence, we obtain the following expression:

3.23 $$p(x_{i,j}|y_{i,1\;:\;j},t_{i,j+1},{\bar{\delta }}_{i,1\;:\;j})\propto p(y_{i,j},t_{i,j+1},{\bar{\delta }}_{i,j}|x_{i,j})p(x_{i,j}|(y_{i,1\;:\;j-1},t_{i,j},{\bar{\delta }}_{i,1\;:\;j-1})),$$

where the latter factor is the prediction distribution for the value of the hidden states at the current time step given all previous observations, while the former updates the posterior distribution of the current hidden states given new evidence. This correction term may be distributed into the product of $p(t_{i,j},{\bar{\delta }}_{i,j}|x_{i,j})$ and $p(y_{i,j}|x_{i,j})$ using the conditional independence assumption. The distributions for these factors can be extracted from equations (3.5) and (3.6), respectively. It was empirically observed that for most cases, the output of equation (3.23) provides a distribution that has a similar shape to a Gaussian distribution, and hence for computational efficiency, we approximate $p(x_{i,j}|y_{i,1\;:\;j},t_{i,j+1},{\bar{\delta }}_{i,1\;:\;j})$ as a Gaussian distribution around its mode with the variance computed from the Hessian matrix, using the Newton–Raphson iterative procedure. Appendix B provides some examples of these empirical observations for a one-dimensional hidden state. These modifications can be incorporated into the RTS smoother with the slight amendments mentioned in the filtering steps to incorporate survival data, having the backward smoothing part of the algorithm remain unchanged.

Having dealt with expectations (3.13)–(3.17), we now turn to equation (3.18). This expectation cannot be computed in closed form, and thus a Laplace approximation can be employed [49, ch. 2]. The detailed derivations of expectations (3.13)–(3.18) are provided in §1 of the electronic supplementary material. The expectations in equations (3.19)–(3.21) are derived using the missing data modifications to the SSM, as explained by [11, ch. 4].

**Maximization step:** This involves finding the parameter values that will set the gradient of the expectation of the complete data log likelihood to zero. The parameters of interest are $\theta =\{{\bar{x}}_{1},{\bar{W}}_{1},A,\overset{˘}{W},V,\gamma ,\alpha \}$. The first five parameters in θ are the state-space parameters, and they have closed-form solutions for their updates given by

3.24 $${\bar{x}}_{1}=\frac{1}{n}\sum_{i=1}^{n}{\hat{\mu }}_{i1}$$

3.25 $${\bar{W}}_{1}=\frac{1}{n}\left(\sum_{i=1}^{n}E[x_{i1}x_{i1}^{⊤}]\right)-{\bar{x}}_{1}{\bar{x}}_{1}^{⊤}$$

3.26 $$\bar{A}=\left(\sum_{i=1}^{n}\sum_{\;j=2}^{m_{i}}E[x_{i,j}^{*}x_{i,j-1}^{⊤}]\right){\left(\sum_{i=1}^{n}\sum_{\;j=2}^{m_{i}}E[x_{i,j-1}x_{i,j-1}^{⊤}]\right)}^{-1}$$

3.27 $$\overset{˘}{W}=\frac{1}{\sum_{i=1}^{n}(m_{i}-1)}\sum_{i=1}^{n}\sum_{\;j=2}^{m_{i}}(E[x_{i,j}^{*}x_{i,j}^{*⊤}]-E[x_{i,j}^{*}x_{i,j-1}^{⊤}]{\bar{A}}^{⊤}-\bar{A}E[x_{i,j-1}x_{i,j}^{*⊤}]+\bar{A}E[x_{i,j-1}x_{i,j-1}^{⊤}]{\bar{A}}^{⊤})$$

3.28 $$V=\frac{1}{\sum_{i=1}^{n}m_{i}}\sum_{i=1}^{n}\sum_{\;j=1}^{m_{i}}(E[y_{i,j}y_{i,j}^{⊤}]-E[y_{i,j}x_{i,j}^{⊤}]C^{⊤}-CE[x_{i,j}y_{i,j}^{⊤}]+CE[x_{i,j}x_{i,j}^{⊤}]C^{⊤}).$$

Note that equations (3.25) and (3.27) require the updated solutions of (3.24) and (3.26) within their formulation, respectively [45]. Furthermore, equations (3.26) and (3.27) are tailored towards the canonical representation of SSM [40]. Slight modifications are required for the general SSM, as shown in §1 of the electronic supplementary material. The updates for the survival parameters γ and α have no closed-form solutions, and therefore we resort to Newton–Raphson iterative procedure, which is formulated as

3.29 $$\left[\begin{array}{c} \gamma^{\left(K\right)}\\ \alpha^{\left(K\right)} \end{array}\right]=\left[\begin{array}{c} \gamma^{\left(K-1\right)}\\ \alpha^{\left(K-1\right)} \end{array}\right]-{\left(H_{f}{|}_{\gamma^{\left(K-1\right)},\alpha^{\left(K-1\right)}}\right)}^{-1}\nabla f{|}_{\gamma^{\left(K-1\right)},\alpha^{\left(K-1\right)}},$$

where *K* represents the iteration of the Newton–Raphson method, ∇f is the gradient vector, *H*_*f*_ is the Hessian matrix, and

3.30 $$\begin{array}{r} \;f\;(\gamma ,\alpha )=\sum_{i=1}^{n}\sum_{\;j=1}^{m_{i}}({\bar{\delta }}_{i,j}\gamma^{⊤}\omega_{i}+{\bar{\delta }}_{i,j}\alpha^{⊤}H{\hat{\mu }}_{i,j}-\tau_{i,j}\exp\left\{\gamma^{⊤}\omega_{i}\right\}\exp\left\{\alpha^{⊤}H{\hat{\mu }}_{i,j}+\frac{1}{2}\alpha^{⊤}H{\hat{\Sigma }}_{i,j}H^{⊤}\alpha \right\}). \end{array}$$

The derivations of all update equations are given in the first section of the electronic supplementary material. After the M step is completed, the algorithm restarts from the E step with the updated parameters, and repeats the process until convergence. The convergence criterion chosen for this algorithm is a difference of less than 5 × 10^−4^ across all parameters. If the algorithm executes 600 EM iterations without reaching this criterion, then it is assumed as failure to converge. Table 1 provides an overview of the estimation procedure for LSDSM. A more detailed summary of the EM algorithm for LSDSM is shown in table 6 in appendix C. The implementation of LSDSM was carried out using MATLAB (v. 9.13.0 (R2022b)).

**Table 1.** Overview of the estimation procedure using the EM algorithm for LSDSM.

1.	Initialize the parameters $\theta^{\left(1\right)}$
2.	For every patient *i*:
3.	→ **Predict** the longitudinal biomarkers using the state space model and **correct** (filter) using the observed longitudinal and survival data
4.	→ **Smoothen** the longitudinal biomarkers trajectories based on the entire observation dataset
5.	**Calculate** the required expectations based on the smoothed results
6.	**Update** the model parameters using these expectations
7.	Repeat lines 2–6 until convergence

## 4. Performance analysis

The predictive performance of survival models is typically assessed through two main criteria, these being the calibration and the discrimination abilities. Calibration states that the model is able to represent and track the data appropriately, while discrimination focuses on concordance, and the model’s ability to discriminate between those that experience the event, to those that do not experience it. In this work, we focus on two performance metrics, these being the dynamic area under the receiver operating characteristic (ROC) curve (AUC), and time-dependent brier score (BS). These are formally explained by Blanche *et al.* [50]. It is claimed that AUC is used for discrimination purposes, while both calibration and discrimination abilities may be captured by BS.

Provided the fact that the proposed framework requires some dynamic data to make individualized predictions, these scores are not analysed at baseline. Hence, we require the use of landmarks and horizons. A landmark is a point in time where the model makes a prediction, allowing the model to use all previous measurements. Horizon is the time span beyond the landmark at which the survival value of the patient is considered for predictions [50]. Landmark time and horizon time are denoted by *t*_*s*_ and *t*_*h*_, respectively. In the case of discrimination, the prediction is whether the patient is expected to experience the event or not at that time, based on some pre-defined threshold. For calibration, this is a simple comparison between the predicted survival value and the observed outcome of the patient at the time of interest.

When assessing the model’s proficiency in tracking longitudinal data, the root mean square error (RMSE) is a widely used method. It provides insights into the average deviation of estimated trajectories from the true biomarker trajectories [51,52]. Hence, we employ RMSE for evaluating longitudinal performance. Additionally, in simulated data where the true survival curve for each patient is known, we employ this metric to assess the model’s calibration capabilities. The equations for the area under the ROC curve, the BS and RMSE, are provided in §2 of the electronic supplementary material.

A recursive solution for dynamic survival predictions using LSDSM is formulated in §3 of the electronic supplementary material. This approach draws upon concepts similar to those used in the E step of the estimation procedure. The subsequent steps provide a concise overview of the solution. At each time step:

1. One-step forward prediction of hidden states $p(x_{i,m_{i}+j}|T_{i}>t_{m_{i}+j},y_{i,1\;:\;m_{i}},{\bar{\delta }}_{i,1\;:\;m_{i}+j-1};\theta )$
2. Correction assuming patient survived the current time step $p(x_{i,m_{i}+j}|T_{i}>t_{m_{i}+j+1},y_{i,1\;:\;m_{i}},{\bar{\delta }}_{i,1\;:\;m_{i}+j};\theta )$
3. One-step forward survival prediction $p(T_{i}>t_{m_{i}+j+1}|T_{i}>t_{m_{i}+1},y_{i,1\;:\;m_{i}},{\bar{\delta }}_{i,1\;:\;m_{i}};\theta )$

The described steps are reiterated until the intended horizon is attained. The initialization of this recursion relies on the final time step of the proposed RTS filter for each patient. It is important to highlight that Steps 2 and 3 are approximated using the Laplace method. In the former, the distribution is approximated as a Gaussian, while in the latter, it is employed to approximate the integral. These approximations are akin to the procedures executed in the proposed modifications of the RTS filter, and can be executed concurrently.

## 5. Simulation study

This section focuses on creating simulations using the LSDSM framework, and assessing the model’s capability to accurately estimate true parameters and effectively track the patient’s true survival curve. The study encompasses an examination of the impact of gradually introducing a higher percentage of ‘missing data’ within the longitudinal biomarkers, i.e. a decreasing number of expected measurement observations per patient. These simulations are constructed based on the LSDSM presented in equations (2.1) and (2.3):

5.1 $$\begin{array}{cc} & x_{i,j+1}=Ax_{i,j}+w_{i,j},\;\;\\ & y_{i,j}=Cx_{i,j}+v_{i,j},\;\;\\ \;\mathrm{and} & h_{i}(t_{i,j})=\exp\left\{\gamma^{⊤}\omega_{i}+\alpha^{⊤}Hx_{i,j}\right\}. \end{array}\}$$

The selection of parameters for the true model is as follows:

5.2 $$A=\left[\begin{array}{cc} 1.46 & -0.48\\ 1 & 0 \end{array}\right],\;\overset{˘}{W}=\left[\begin{array}{c} 0.04 \end{array}\right],\;V=\left[\begin{array}{c} 0.25 \end{array}\right],\gamma =\left[\begin{array}{c} 2.5\\ -0.75 \end{array}\right]\;\mathrm{and}\;\alpha =\left[\begin{array}{c} -1.25 \end{array}\right].$$

While the following parameters are assumed fixed and known:

5.3 $$C=\left[\begin{array}{cc} 1 & 0 \end{array}\right],\;{\bar{x}}_{1}=\left[\begin{array}{c} 10\\ 10 \end{array}\right],\;{\bar{W}}_{1}=\left[\begin{array}{cc} 1 & 0\\ 0 & 1 \end{array}\right]\;\mathrm{and}\;H=\left[\begin{array}{cc} 1 & 0 \end{array}\right].$$

This setup incorporates a solitary biomarker trajectory, two states derived from recent history, an intercept value for the baseline hazard, a baseline covariate sampled from a normal distribution N(0,1), and a direct influence of the current true biomarker value on the hazard function. It is noteworthy that due to the utilization of a canonical representation of the SSM, $\overset{˘}{W}$ is a scalar denoting the disturbance variance in the AR(2) model. The choice of variances ensures that both the disturbance and the measurement error possess zero mean and standard deviations of 0.2 and 0.5, respectively.

This setup mirrors real-world scenarios where a patient’s health might gradually decline over time, with occasional disturbances influencing either improvement or further deterioration in their health status, alongside potential measurement errors linked to recording biomarker observations. The trajectory dynamics resemble those observed in the walking distance trajectories of patients with PAH.

To simulate scenarios resembling real-world data situations where observations are frequently missing (due to irregular clinic appointments, patient absenteeism etc.), a certain percentage of observations are randomly assigned as not a number (NaN) values. The survival times and event indicators for each patient are calculated using the inverse transform sampling method as outlined by Walke [53]. This procedure emulates the piecewise exponential survival function, adhering to the stated assumptions. The survival status of each patient is assessed at every time step. Censoring times were drawn from a uniform distribution $C_{i}^{*}∼U(10,50)$. The maximum observational study duration for each patient is capped at 30 time steps, denoted as $C_{i}=\min(30,C_{i}^{*})$. This can be likened to bi-monthly follow-ups over a span of 5 years. This simulation setup results in approximately 49% of patients experiencing the event, with an average survival time of around 21 time points.

The training dataset comprises of 500 simulated patients. The missing observation percentages are set at 0%, 25%, 50% and 75%. For each configuration, 100 simulations are generated. Table 2 presents summary statistics for the estimated parameters within this simulation study. This table records the average values across all simulations for each configuration, alongside the sample standard deviation. Notably, the model successfully converges in all runs during these simulations. On average, convergence is achieved within 26, 11, 17 and 50 EM iterations for the 0%, 25%, 50% and 75% missing observations configurations, respectively. It is observed that all true parameter values lie within 1 standard deviation of the estimated parameter samples, except for the survival parameters in the 75% missing observations configuration, where bias is evident. Therefore, it is advisable to select the Δ*t* hyperparameter, signifying the time step for the SSM, such that the fraction of missing longitudinal observations remains within 50%. Histograms depicting the estimated parameters’ distributions are presented in figure 7 in appendix D.

**Table 2.** Parameter estimation for the simulation study, showing the average and sample standard deviation across several configurations.

		configuration—missing observations (expected number of measurements)			
parameter	true value	0% (*E*[*m*_*i*_] = 21)	25% (*E*[*m*_*i*_] = 16)	50% (*E*[*m*_*i*_] = 11)	75% (*E*[*m*_*i*_] = 6)
$A_{11}$	1.46	1.4490 ± 0.0145	1.4568 ± 0.0127	1.4671 ± 0.0154	1.4602 ± 0.0190
$A_{12}$	−0.48	−0.4695 ± 0.0141	−0.4770 ± 0.0123	−0.4869 ± 0.0149	−0.4804 ± 0.0184
$\overset{˘}{W}$	0.04	0.0425 ± 0.0026	0.0410 ± 0.0020	0.0391 ± 0.0027	0.0427 ± 0.0043
*V*	0.25	0.2481 ± 0.0046	0.2484 ± 0.0046	0.2501 ± 0.0065	0.2440 ± 0.0126
$\gamma_{s1}$	2.5	2.5329 ± 0.2543	2.5062 ± 0.2585	2.4362 ± 0.2421	1.9782 ± 0.1889
$\gamma_{s2}$	−0.75	−0.7596 ± 0.0651	−0.7563 ± 0.0666	−0.7467 ± 0.0691	−0.6992 ± 0.0662
*α*_*s*1_	−1.25	−1.2555 ± 0.0619	−1.2496 ± 0.0628	−1.2347 ± 0.0584	−1.1393 ± 0.0447

We also evaluate the RMSE of both the longitudinal and survival trajectories to assess the model’s ability to accurately track these crucial indicators. These assessments are conducted on both the training and testing data to ensure that the estimated model does not suffer from overfitting, and can generalize to unseen data. Furthermore, we examine the first hidden state and the corresponding survival curve for each simulation. Additionally, we calculate the RMSE for the curves using the true and estimated model parameters for comparison. This is done to mitigate the RMSE variation observed across all simulations due to the inherent stochasticity. The results are summarized in table 3. It is evident that the RMSE values obtained using the estimated model closely resemble those obtained with the true model parameters when tracking longitudinal and survival data, and thus are within an acceptable level.

**Table 3.** Average RMSE across all simulations for the training and testing datasets, for the estimated and the true models compared to the simulated true hidden values and survival trajectories, across several configurations.

	configuration (missing observations)							
	0% (*E*[*m*_*i*_] = 21)		25% (*E*[*m*_*i*_] = 16)		50% (*E*[*m*_*i*_] = 11)		75% (*E*[*m*_*i*_] = 6)	
trajectory	true	est.	true	est.	true	est.	true	est.
$\mu_{1}$ (train)	0.3358	0.3359	0.4024	0.4024	0.5072	0.5072	0.7052	0.7057
$\mu_{1}$ (test)	0.3359	0.3360	0.4024	0.4025	0.5079	0.5082	0.7059	0.7069
survival (train)	0.0274	0.0290	0.0331	0.0345	0.0426	0.0438	0.0613	0.0623
survival (test)	0.0269	0.0287	0.0327	0.0341	0.0419	0.0432	0.0609	0.0621

Notably, in the 75% missing observations scenario, even when the correct parameters are employed, the model still exhibits difficulty in accurately tracking the survival curve, with an average error exceeding 5% in survival probability. This is due to the larger uncertainty in the true hidden state values with more sequential missing observations. Despite the apparent bias in the survival parameters for the estimated model in this scenario, the RMSE values for the survival trajectories are nearly identical to those obtained using the true model. As evidenced in figure 8 in appendix E, with the limited number of observed measurements, multiple solutions exist with different parameter values providing similar results, despite the observed bias in the survival parameters.

These observations underscore the importance of selecting the hyperparameter Δ*t* appropriately, with consideration for maintaining the percentage of missing measurements around 50%.

## 6. Analysis on a real dataset

This work is primarily motivated by the need for improved survival analysis for patients with PAH. PAH is a rare and life-threatening condition characterized by vascular proliferation that results in elevated pressure in the pulmonary artery [10]. This often leads to reduced cardiac output, which in turn manifests as limited exercise capacity in patients [9,10]. The dataset employed in this study was collected at the Sheffield pulmonary vascular disease unit. Patients at this unit undergo regular follow-ups, typically recommended at intervals of three to six months [54]. Among the various tests conducted, the exercise test is frequently administered, with a focus on either the 6-minute walk test or the incremental shuttle walk test. Our investigation primarily centres on the latter test, examining how the trajectories of walking distances across several exercise tests relate to the survival of PAH patients.

Currently, the prevailing method for assessing the risk of death in PAH patients involves using a risk score, such as REVEAL 2.0 risk score and the European Society of Cardiology/European Respiratory Society (ESC/ERS) risk-assessment model [54]. However, these scores do not account for longitudinal trends in relevant biomarkers. Some initial efforts have been made to incorporate these trends, but they are inherently limited, often involving simple checks on changes in walking distance over the past year and defining thresholds to adjust patient risk [10]. To the best of our knowledge, there has been no prior study that jointly models longitudinal and survival data within this dataset.

The data were collected at Sheffield Pulmonary Vascular Disease Unit and include a total of 5391 patients. This dataset encompasses patients with pulmonary hypertension beyond just those classified as having PAH. After specifically selecting PAH patients and ensuring that each patient had undergone at least two exercise tests, the dataset was narrowed down to 1105 patients. On average, this cohort was approximately 59.5 years old at the time of diagnosis ( ±15.5 years), with an average follow-up period of 4.25 years (±2.79 years). Among these patients, 376 (34%) experienced the event of interest, which in this case is death, during the observed 10-year period. The average number of measurements per patient in this dataset was approximately 6.6 (±4.1) observations. The median time between visits was six months, with a mean period of 7 (±5.9) months. The majority of patients were females, accounting for a total of 788 (71.3%). This is an expected observation given that prevalence in females is higher [54]. A large portion of the patients were classified in World Health Organization (WHO) functional classes III and IV (1043), leaving only 9.4% of the patients within classes I or II.

Our objective is to identify patients who face a higher risk of death. This enables clinicians to anticipate and provide personalized treatment to those at greater risk, potentially prolonging their survival time. In our model, we consider age, sex and a binary variable indicating whether patients belong to WHO functional classes I and II or III and IV as baseline covariates. We model the walking distance as the longitudinal trajectory using the AR(2) configuration described in previous sections. Upon initial inspection of the longitudinal data, the dynamics appear relatively straightforward, prompting the selection of a low AR order. Various AR orders were evaluated, and it was found that the second order yielded superior results while maintaining parsimony. The observed walking distance ranges from 0 to 1020 m, truncated at 1000 m for easier interpretability of coefficients, and normalized to a scale ranging from 0 to 1. This truncation affected only 21 data points. Hence, a normalized value of 0.5 now corresponds to 500 m in the response variable rather than 510 m. This simplifies interpretation when analysing hazard ratios. Our SSM consists of two states, with one tracking the other. We assume that the hazard function is influenced by the baseline covariates and only the current value of the true walking distance, with the lagging state not affecting a patient’s survival, thus H=[1 0]. Alternative configurations were explored, yet none yielded improvements in the results. Therefore, for the sake of parsimony, this choice was ultimately selected.

We divided the available data such that 70% of the patients are in the training dataset, resulting in 773 patients for training, while the remaining 332 patients constitute the testing dataset for evaluating the model’s performance on unseen data.

The estimated parameters, denoted as θ, encompassed the following variables: $\left\{{\bar{x}}_{1},{\bar{W}}_{1},A,\overset{˘}{W},V,\gamma ,\alpha \right\}$. The matrix C was held fixed at [1 0], indicating a direct linkage between the observed measurements and the true underlying biomarker value with some associated measurement error. The hyperparameter Δ*t* was configured to a duration of six months, aligning with the median time between visits and falling within the recommended timeframe for regular follow-up of PAH patients. This resulted in approximately 45% missing observations and an average of five observations per patient, which falls within the recommendation outlined in the simulation study of retaining missing observations within 50%. The model achieved convergence within 23 EM iterations. The deduced parameter values are as follows:

6.1 $$\begin{array}{cc} & A=\left[\begin{array}{cc} 1.39 & -0.40\\ 1 & 0 \end{array}\right],\;\overset{˘}{W}=\left[\begin{array}{c} 5.4\times 10^{-4} \end{array}\right],\;V=\left[\begin{array}{c} 1.7\times 10^{-3} \end{array}\right],\\ & {\bar{x}}_{1}=\left[\begin{array}{c} 0.19\\ 0.16 \end{array}\right],\;{\bar{W}}_{1}=\left[\begin{array}{cc} 0.018 & 0.014\\ 0.014 & 0.022 \end{array}\right],\;\gamma =\left[\begin{array}{c} -7.05\\ 0.0337\\ 0.51\\ 1.03 \end{array}\right]\;\mathrm{and}\;\alpha =\left[\begin{array}{c} -8.23 \end{array}\right]. \end{array}\}$$

From these parameter values, we can infer that the population’s walking distance tends to gradually decrease over time. The transition matrix further reveals that, on average, the rate of change decreases to approximately 0.4 times the rate of change at the previous time point. This suggests that while the walking distance is gradually declining, the rate of decline decelerates over time, indicating a trend towards more stability in the walking distance. The model estimates the process and measurement error standard deviations to be 0.023 and 0.041, roughly equivalent to 23 and 41 m, respectively. For a new patient with no initial values, the model assumes an approximate starting point of 190 m, with a standard deviation of 135 m. While this standard deviation is relatively large, it reflects the lack of prior information about this particular patient. Appendix G shows some examples of patient longitudinal observations together with their expected leading hidden state trajectories.

**Figure 3.**
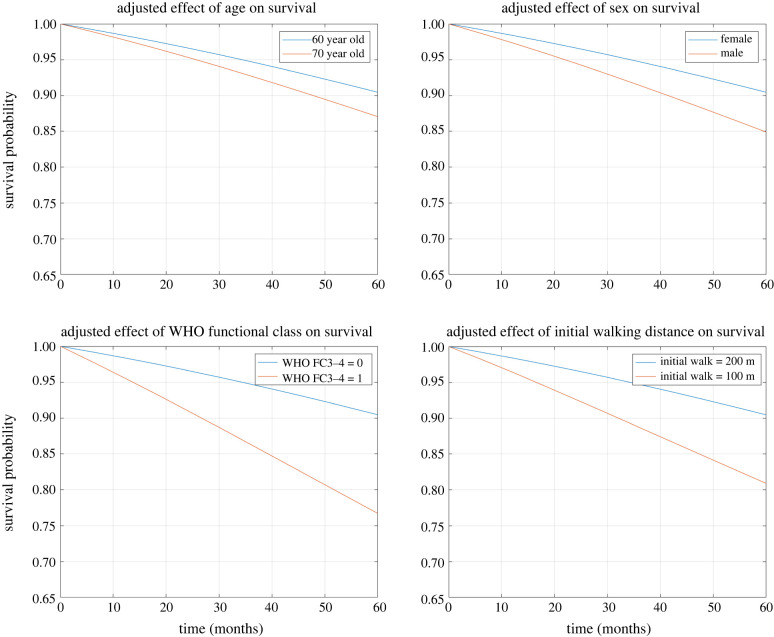
Survival trajectories for patients with different age, sex, WHO functional class and initial walking distance.

The patient’s age (*γ*_1_ = 0.0337), sex (*γ*_2_ = 0.51) and membership in WHO functional classes III or IV (*γ*_2_ = 1.03) make proportionally high contributions to the hazard function. Specifically, a patient who is 10 years older has a hazard ratio of 1.4, while being male increases the hazard, with a hazard ratio of 1.67. Belonging to the more severe WHO functional classes results in a hazard ratio of 2.79 compared to those in lower functional classes. Additionally, we observe a notable effect of the true walking distance on the hazard function (*α* = −8.23), with a hazard ratio of 0.44 for a patient who walks an extra 100 metres. In summary, we illustrate the impact of baseline covariates and initial walking distance on a patient’s survival with four plots in figure 3. These findings confirm the effects of covariates on the hazard, aligning with risk scores for PAH [8].

**Figure 4.**
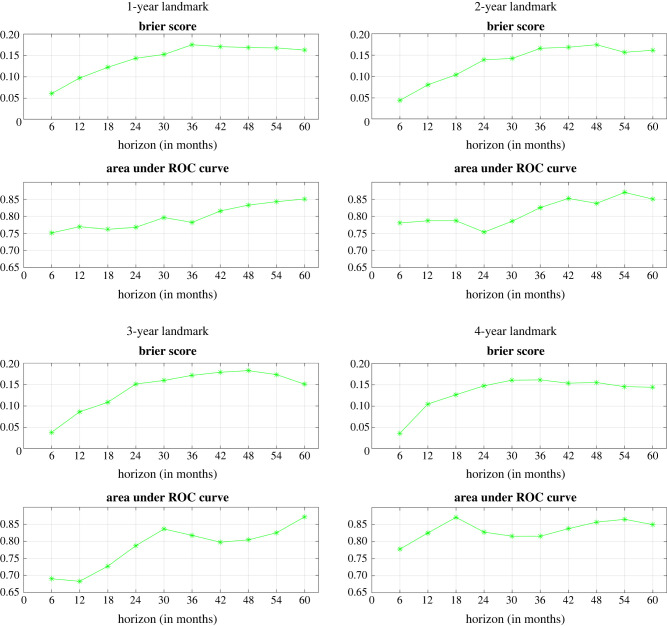
Performance metrics across different landmarks for a maximum horizon of 5 years.

Next, we assess the model’s performance on the test dataset. We evaluate the BS and the AUC for various horizons and landmarks. Specifically, we examine landmarks at 1, 2, 3 and 4 years, and for each of these landmarks, we analyse evenly spaced horizons ranging from 6 months to 5 years. The results are presented in figure 4. From these plots, it is evident that as the landmark time increases, the model’s accuracy also improves. Notably, we observe a slightly lower average BS and a higher AUC for the 4-year landmark compared to the 1- and 2-year landmarks. This implies that predictions become more accurate as patients are tracked over longer durations, which aligns with inherent expectations.

Finally, we assess the model’s performance in comparison to a risk score developed for risk stratification of PAH patients. Since our dataset predominantly employs the incremental shuttle walk test rather than the 6-minute walk test, we compare our model with the approach by Billings *et al.* [10], which uses only the walking distance as a prognostic covariate and employs landmarking techniques for evaluation at different times.

Billings *et al.* [10] also employ thresholding techniques to classify patients and evaluate sensitivity and specificity values for various thresholds, which are then used to compute the area under the ROC curve. To generate this curve, we consider threshold values at 10 m intervals, ranging from 0 to 1000 m. We perform these comparisons across the same landmarks as in the previous analysis, ensuring that only patients who underwent an exercise test within two months prior to the landmark time are included, reducing the effective test sample size. We evaluate the performance metric for horizons of 1, 2 and 3 years, as was performed by Billings *et al.* [10]. This was performed using R (v. 4.3.1).

To ensure a fair comparison, we retrain LSDSM without incorporating any baseline covariates. We also employ thresholding techniques to compute the AUC; however, these thresholds are based on the forecasted survival values at the horizon of interest rather than the actual walking distance. Furthermore, the model is limited to obtaining performance metrics solely for patients used in the comparison model. In order to prevent a significant reduction in the size of the testing sample, we divided the data to allocate 50% of the patients to the testing dataset, which necessitated retraining LSDSM on a smaller dataset. This led to minor changes in the parameter values.

**Figure 5.**
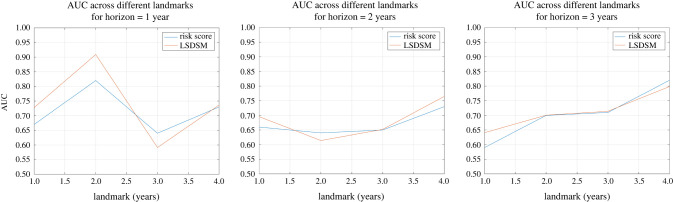
AUC across different landmarks and horizons for the risk score [10] compared to LSDSM.

**Figure 7.**
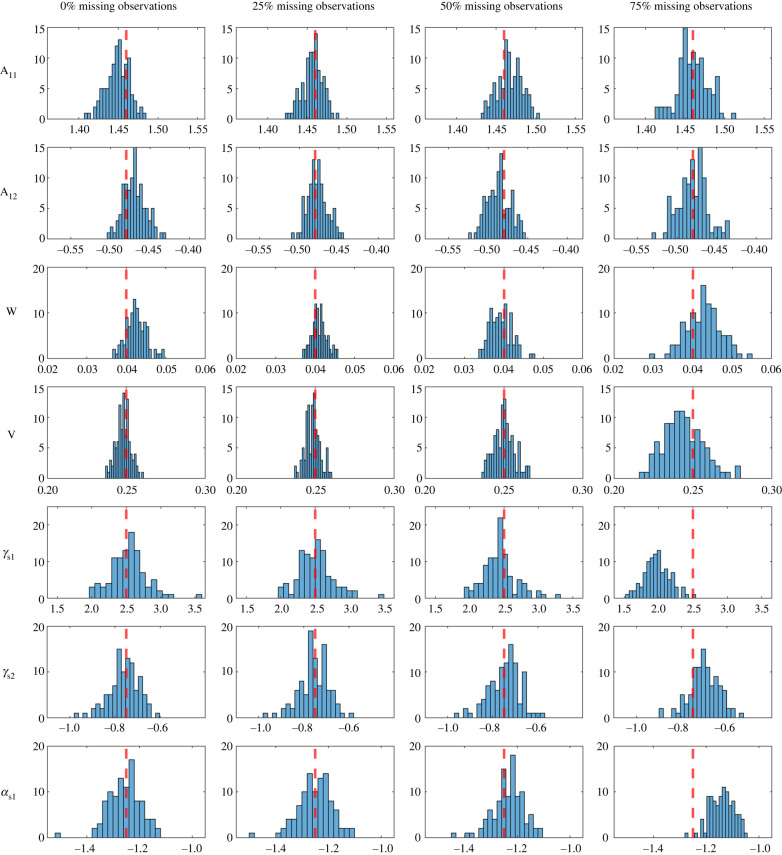
Histograms of estimated parameters for every configuration of 0%, 25%, 50% and 75% missing values. Red vertical line indicates true value.

**Figure 8.**
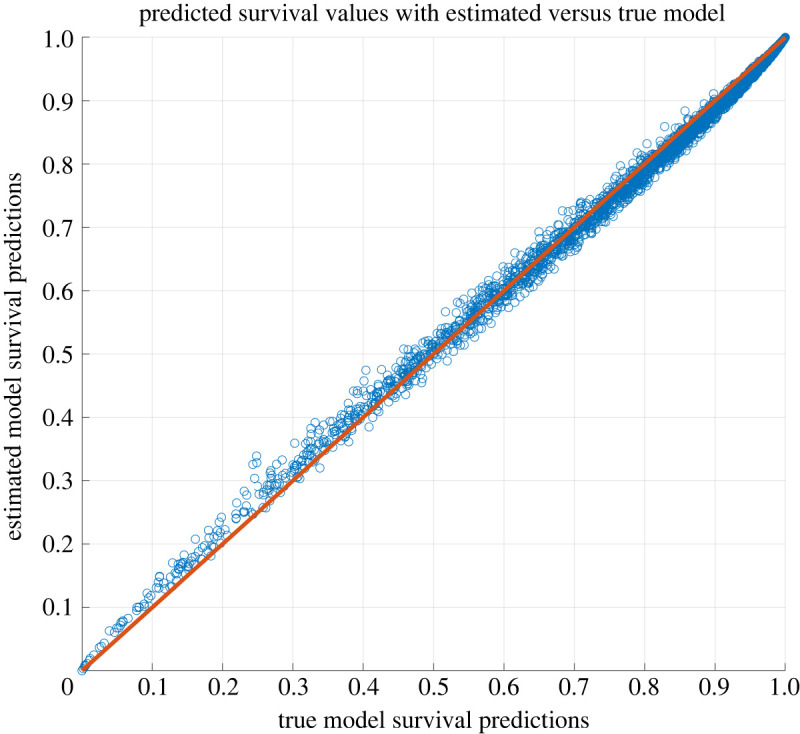
A scatter plot showing the predicted survival values using the estimated model against the true model for a simulation with biased estimated parameters.

The results are illustrated in figure 5. It is evident that in nearly all scenarios, both methods produce comparable outcomes, with a slight advantage in favour of LSDSM. Notably, LSDSM outperforms the risk score method in all configurations except one within the 1-year horizon. This is significant because the 1-year horizon is often the most clinically relevant for healthcare providers treating PAH patients [8,54]. This finding underscores the promising potential of employing a framework jointly modelling longitudinal and survival data for dynamic survival predictions in PAH patients, as opposed to static models that rely on landmarking. An additional advantage of the proposed model is its efficiency in generating results. Unlike risk scores, which necessitated the creation of 12 separately trained models, our model achieved all results using a single integrated framework. However, it is crucial to exercise caution when interpreting these results due to the limited number of patients available under the stringent configuration made for a fair comparison. Specifically, there were only 117, 85, 67 and 40 patients for landmarks at 1, 2, 3 and 4 years, respectively, within the test dataset.

## 7. Discussion

The LSDSM presents a unique capability to concurrently monitor and forecast longitudinal and survival data using an SSM. It leverages system identification techniques offered by SSM while integrating survival information into state and parameter estimation for effective hazard modelling. This framework provides interpretability for the dynamics through coefficients that describe the evolution of biomarkers and their interdependencies. The incorporation of SSM into the longitudinal process can be especially advantageous when tracking physiological trajectories that follow differential or difference equations over time [55].

The simulation studies provided valuable insights into the performance of the proposed model under varying percentages of missing observations. The simulations were set up with different missing data configurations rather than adjusting time step values to understand bias effects without changing parameters. This is effectively equivalent to changing the time step hyperparameter, where increasing values of missing data may correspond to a shorter time step. Thus, the time step choice was also indirectly evaluated here. It was observed that accurately estimating survival parameters becomes challenging when dealing with 75% missing observations in the longitudinal trajectory. Therefore, it is advisable that when selecting the hyperparameter Δ*t*, which determines the time step of the SSM, the percentage of missing observations should be kept within 50%. Another supporting observation is that the average RMSE of the survival trajectories over time remained within a 5% error margin for configurations with 50% or less missing data. By contrast, this margin was exceeded when dealing with the 75% missing data configuration, even when using the true parameter values for LSDSM. These findings offer valuable guidance for choosing the hyperparameter when applying LSDSM to real-world data.

In the practical application of LSDSM to predict survival probabilities for patients with PAH, who typically undergo multiple exercise tests during their follow-up and treatment, the longitudinal data were derived from the walking distance recorded in these tests. The analysis revealed that, on average, the walking distance trajectory gradually decreased over time, resulting in a continuous increase in the hazard rate. Additionally, factors such as age, sex and WHO functional classes were found to influence the hazard, aligning with previous findings by other researchers in this field [8,54]. Furthermore, it was confirmed that longer observation periods for patients lead to improved survival predictions, supporting the intuitive notion that more extended patient histories yield better predictions.

Moreover, LSDSM was compared to a risk score that relied solely on walking distance as a prognostic factor. The results demonstrated that LSDSM holds the potential to enhance the predictive accuracy compared to this risk score. Nevertheless, further research is warranted to validate this hypothesis, potentially through a comparison with a more comprehensive risk score that necessitates a richer dataset.

The data in clinical settings, such as those for patients with PAH, are often sparse due to infrequent patient visits. However, this sparsity does not necessarily mean missing information. Missing information arises as measurements within discretized time intervals aggregate. In PAH datasets, instances where two measurements fall within the same time interval are rare, and when they do occur, they typically have similar values, minimizing information loss. Continuous-time survival models are often based on conventional basis functions, which can interpolate between observations. System dynamics can be represented either in basis function form or in state space parametrized forms, there being mathematical equivalence of continuous-time models with their discrete counterparts in linear dynamical systems. The equivalence only applies at the discrete-time points at which there are deemed to be observations or where predictions are to be made. This would be natural if the patients are typically monitored on a regular basis, as is suggested in the PAH guidelines for optimal care. Hence the proposed time interval of six months within this application for LSDSM [54].

This model exhibits versatile applicability across various domains. It can serve as a potent tool for screening individuals in at-risk populations who undergo recurrent assessments over time. Furthermore, it finds utility in evaluating treatment responses to specific therapies, leveraging longitudinal follow-up data to gauge efficacy. Additionally, this model contributes to the fine-tuning of mortality predictions, which can be instrumental in guiding counselling and treatment decisions. Lastly, the LSDSM holds promise in the realm of patient prioritization for transplantation, exemplified in cases like PAH. By assessing the risk of mortality of the patients, it aids in the selection of candidates for urgent transplant procedures. This approach stands in contrast to existing empirical methods that rely on disease severity, often lacking a structured risk assessment.

If the AR order remains constant, then the number of parameters grows quadratically with an increasing number of biomarker trajectories. This implies that, as expected, a large dataset may be necessary for highly intricate models. However, it is worth noting that the computational time for the estimation procedure should remain relatively stable, owing to the computational efficiency of the RTS smoother, which is a significant advantage of LSDSM. The number of parameters can be seen in §4 of the electronic supplementary material. To reduce the number of estimated parameters, expert knowledge can be leveraged. If certain biomarkers are known to be statistically independent of others, a neighbourhood structure can be introduced. This allows for the retention of relevant parameters equivalent to zero throughout the estimation process, as demonstrated by Dewar & Kadirkamanathan [40].

The proposed model has a few limitations. First, LSDSM assumes a regular time series, and the approximations made may result in reduced accuracy. More specifically, since it bins observations into fixed intervals, occasional measurements between smaller time intervals may being disregarded. This also results in the additional hyperparameter of choosing the time step Δ*t*. Second, LSDSM as derived here assumes a constant baseline hazard function, restricting the flexibility granted by other functions. However, this variation is implicit through the variation of the hidden states. That being said, extensions to include time-varying population mean survival curves can be readily made. Third, the proposed model only accounts for linear dynamics, limiting the complexity that can be captured in the longitudinal process. In this regard, the estimation procedure was greatly simplified, allowing us to use a more computationally efficient estimation method. Nonlinear dynamics may be included on this established framework; however, linear models do retain the capability to capture the approximate dynamics in the sparse data with fewer parameters. Fourth, only controllable and observable biomarker dynamics can be captured by the LSDSM approach. Incorporating unobservable and/or uncontrollable biomarkers will require strong assumptions and *a priori* knowledge of the mechanistic model, as carried out by Desmée *et al.* [56] with the JM. Fifth, this model assumes that all patients follow a single population dynamics with some disturbances. While individualized predictions are still possible for new patients, the heterogeneity within the patient dataset may not allow us to extract the exact disturbances within every patient that directly reveal the comprehensive deviations from population. With limited data, this assumption helps with capturing the common effects that are seen across this cohort of patients. Lastly, another limitation emerges from the simulation study performed, where it consists of a single experimental setup covering a single biomarker trajectory based on an AR(2) process, with a single baseline covariate.

Future endeavours could address several of these limitations. For instance, exploring alternative parametric functions, such as the Weibull distribution, for the baseline hazard function could be considered. This would involve adjusting the hazard function accordingly and determining equations that optimize the expectation of the complete data log likelihood with respect to these new parameters. Incorporating nonlinear longitudinal biomarkers might involve adapting the dynamics and observation equations to suit the anticipated distributions. This allows the framework to model more complex patterns and perhaps improve longitudinal and survival predictions. Moreover, the population variation may be accomplished by introducing further randomized effects into the model. It is also worth noting that the current model naturally accommodates the inclusion of additional longitudinal biomarkers by adapting the model parameters. The LSDSM approach could uncover inherent correlations among these biomarkers. However, this study did not delve into the analysis of multiple biomarkers, which is an avenue for future exploration and can be pursued effortlessly. Future work may also address the simulation study limitation by analysing more complex setups, including multiple longitudinal biomarkers, and additional baseline covariates affecting the hazard function.

LSDSM presents a promising avenue for jointly modelling longitudinal and survival data. Exploring the application of LSDSM to diverse datasets, particularly those featuring regular time-series data from sources like wearable technology, holds substantial research potential and would be a compelling avenue for further investigation. The estimation procedure is developed within a maximum-likelihood framework. Our proposed model has been applied to both real-world and synthetic datasets, yielding encouraging results in terms of survival predictions for PAH patients. It exhibits an advantage over a conventional risk score, the prevalent method used for PAH patient survival analysis. Furthermore, LSDSM offers flexibility in terms of complexity, although this scalability may necessitate larger datasets for accurate parameter estimations. In conclusion, LSDSM provides an alternative approach to the JM for longitudinal and survival data, explaining dynamics as a function of past true biomarker values. This unique perspective opens up significant opportunities for further improvements, drawing from the extensive research available in SSMs.

## Figures and Tables

**Figure 6.**
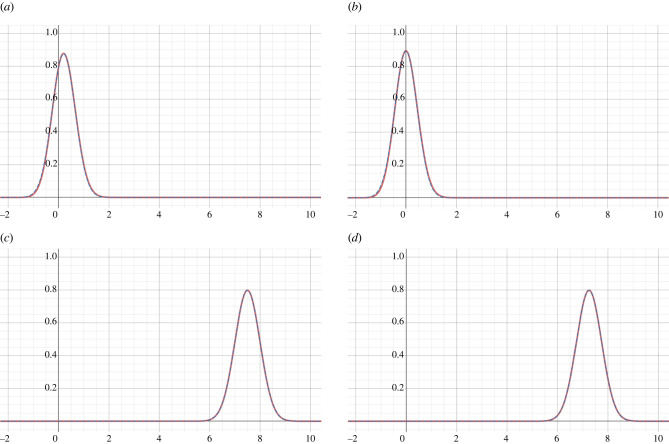
Four configurations illustrating the true (red) distribution alongside its approximated (dashed blue) Gaussian distribution. (*a*) Config. 1: $y_{i,j}={\overset{ˇ}{\mu }}_{i,j}^{\left(pred\right)}=0,\delta_{i,j}=0$. (*b*) Config. 2: $y_{i,j}={\overset{ˇ}{\mu }}_{i,j}^{\left(pred\right)}=0,\delta_{i,j}=1$. (*c*) Config. 3: $y_{i,j}={\overset{ˇ}{\mu }}_{i,j}^{\left(pred\right)}=7.5,\delta_{i,j}=0$. (*d*) Config. 4: $y_{i,j}={\overset{ˇ}{\mu }}_{i,j}^{\left(pred\right)}=7.5,\delta_{i,j}=1$.

**Figure 9.**
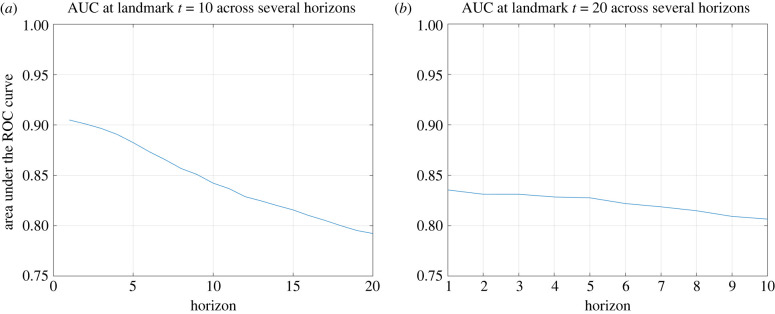
Averaged AUC across several horizons for landmarks *t* = 10 (*a*) and *t* = 20 (*b*).

**Figure 10.**
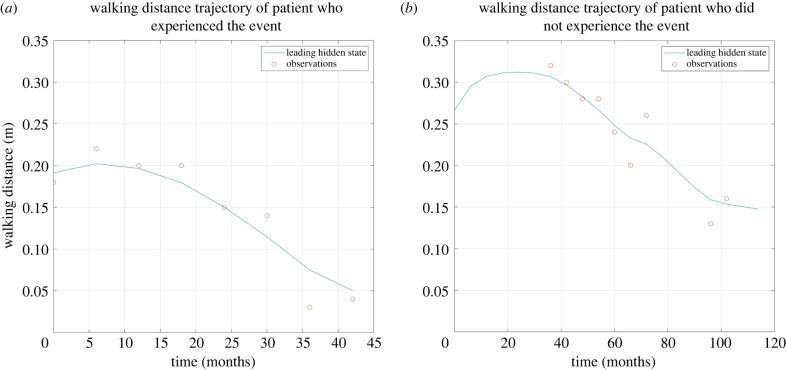
Example data illustrating the longitudinal trajectories of two patients. (*a*) A patient who experienced the event of interest, while (*b*) a patient who did not.

## Data Availability

The de-identified data are available on the University of Sheffield’s data repository (ORDA): https://figshare.com/s/10d3968fb4a9c166b942 [57]. The data are provided in electronic supplementary material [58].

## Appendix A. Table of notation

**Table 4.** Table of notation for the proposed model framework.

$T_{i}^{*}$	survival time for individual *i*
*C*_*i*_	censoring time for individual *i*
$T_{i}=\min(T_{i}^{*},C_{i})$	observed time for individual *i*
$\delta_{i}=I(T_{i}^{*}\leq C_{i})$	event indicator for individual *i* (=1 if event occurs, 0 otherwise)
*m*_*i*_	number of biomarker measurements for individual *i* across the observation period
*M*	autoregressive order number
*m*_*y*_	number of unique recorded longitudinal biomarkers
*m*_*x*_ = *M* × *m*_*y*_	number of hidden states considered in state space model
*n*	number of individuals in the study
$t_{i}=\{t_{i,j}\;:\;t_{i,j}\leq T_{i},j=1,\ldots ,m_{i}\}$	set of timings of biomarker measurements for individual *i*
$y_{i,j}=y_{i}(t_{i,j})\in R^{m_{y}\times 1}$	biomarker measurements vector for patient *i* at time *t*_*i*,*j*_
$Y_{i}=\{y_{i,j}\;:\;j=1,\ldots ,m_{i}\}$	set of all biomarker measurements for individual *i*
$x_{i,j}=x_{i}(t_{i,j})\in R^{m_{x}\times 1}$	true biomarker values for patient *i* at time *t*_*i*,*j*_
$X_{i}=\{x_{i,j}\;:\;j=1,\ldots ,m_{i}\}$	set of true underlying biomarker values for individual *i*
Δ*t*	fixed time step for the state space model such that *j*Δ*t* = *t*_*i*,*j*_
*τ*_*i*,*j*_	time step for period *j* of patient *i*: $\tau_{i,j}=\Delta t,\;\;\forall j\neq m_{i}$, and $\tau_{i,m_{i}}=T_{i}-m_{i}\Delta t$
$w_{i,j}\in R^{m_{x}\times 1}$	model errors vector for patient *i* at time *t*_*i*,*j*_
$v_{i,j}\in R^{m_{y}\times 1}$	biomarker measurement errors vector for patient *i* at time *t*_*i*,*j*_
$A\in R^{m_{x}\times m_{x}}$	state matrix governing the dynamics in the state space model
$C\in R^{m_{y}\times m_{x}}$	observation matrix dictating the observed states in the state space model
$W\in R^{m_{x}\times m_{x}}$	covariance matrix of the disturbance term
$V\in R^{m_{y}\times m_{y}}$	covariance matrix of the biomarker measurement error
${\bar{x}}_{1}\in R^{m_{x}\times 1}$	initial conditions vector of the hidden states
${\bar{W}}_{1}\in R^{m_{x}\times m_{x}}$	covariance matrix for the initial conditions of the hidden states
*h*_*i*_ (*t*)	hazard function for individual *i*
$\omega_{i}\in R^{m_{\gamma }\times 1}$	baseline covariates vector for individual *i*
$\gamma \in R^{m_{\gamma }\times 1}$	corresponding coefficients vector for effects of baseline covariates on survival
$\alpha \in R^{m_{\alpha }\times 1}$	parameters relating the longitudinal and survival processes
$H\in R^{m_{\alpha }\times m_{x}}$	matrix taking a linear combination of the hidden states to relate to the hazard function
$D_{i}^{\left(o\right)}=(T_{i},\delta_{i},Y_{i},t_{i},\omega_{i})$	observed data for individual *i*
$D_{i}^{\left(c\right)}=(T_{i},\delta_{i},Y_{i},t_{i},\omega_{i},X_{i})$	complete data of LSDSM for individual *i*
$\theta =\{{\bar{x}}_{1},{\bar{W}}_{1},A,W,V,\gamma ,\alpha \}$	parameter set to be estimated for the proposed joint model

## Appendix B. Gaussian-approximated filter distribution

Table 5 illustrates four instances of varied configurations. Figure 6 displays the true distribution of equation (3.22) alongside a Gaussian approximation achieved through Laplace approximation using the Newton–Raphson method. It is evident that the approximation closely resembles the true distribution, exhibiting only slight differences, primarily noticeable near the tails of the distribution.

**Table 5.** Four configurations used to assess the performance of approximating the filter posterior distribution as a Gaussian.

configuration	*y*_*i*,*j*_	${\overset{ˇ}{\mu }}_{i,j}^{\left(pred\right)}$	*δ*_*i*,*j*_
1	0	0	0
2	0	0	1
3	7.5	7.5	0
4	7.5	7.5	1

It was observed through empirical analysis that, in many instances, the outcome of equation (3.22) exhibits a distribution with a shape resembling that of a Gaussian distribution. Furthermore, this equation on expansion, bears a resemblance to a negative quadratic in the exponent, reinforcing the notion that this function behaves akin to a normal distribution. Therefore, for computational efficiency, we approximate $p(x_{i,j}|y_{i,1\;:\;j},t_{i,j+1},\delta_{i,1\;:\;j})$ as a Gaussian distribution centred around its mode, with the variance determined from the inverse of the Hessian matrix using the Newton–Raphson iterative method. Although this approximation involves a slight sacrifice in accuracy, it allows for the advantageous computational simplicity of working with Gaussian distributions, streamlining the state estimation process.

## Appendix C. Expected maximization algorithm for LSDSM

See table 6.

**Table 6.** Summary of EM algorithm for LSDSM.

1.	Initialize parameters $\theta^{\left(1\right)}$ and *k* = 1
2.	While convergence criteria not met:
3,	→ *k* = *k* + 1
4.	→ **E Step**—For every patient *i*:
5.	→ For every time step *j* = 1, …, *m*_*i*_ (Filtering):
6.	→ Predict the current hidden state values: $p(x_{i,j}|y_{i,1\;:\;j-1}^{\left(O\right)},t_{i,j},{\bar{\delta }}_{i,1\;:\;j-1})\approx N({\overset{ˇ}{\mu }}_{i,j}^{\left(pred\right)},P_{i,j-1})$:
7.	→ If *j* = 1 (Initialisation):
8.	→ ${\overset{ˇ}{\mu }}_{i,1}^{\left(pred\right)}={\bar{x}}_{1}$
9.	→ $P_{i,0}={\bar{W}}_{1}$
10.	→ If *j* = 2, …, *m*_*i*_:
11.	→ ${\overset{ˇ}{\mu }}_{i,j}^{\left(pred\right)}=A{\tilde{\mu }}_{i,j-1}$
12.	→ $P_{i,j-1}=A{\tilde{\Sigma }}_{i,j-1}A^{⊤}+W$
13.	→ Correction step using the current longitudinal data: $p(x_{i,j}|y_{i,1\;:\;j}^{\left(O\right)},t_{i,j},{\bar{\delta }}_{i,1\;:\;j-1})\approx N({\overset{ˇ}{\mu }}_{i,j},{\overset{ˇ}{\Sigma }}_{i,j})$:
14.	→ $K_{i,j}^{*}=P_{i,j-1}C^{*⊤}{\left(C^{*}P_{i,j-1}C^{*⊤}+V^{*}\right)}^{-1}$
15.	→ ${\overset{ˇ}{\mu }}_{i,j}={\overset{ˇ}{\mu }}_{i,j}^{\left(pred\right)}+K_{i,j}^{*}(y_{i,j}^{*}-C^{*}{\overset{ˇ}{\mu }}_{i,j}^{\left(pred\right)})$
16.	→ ${\overset{ˇ}{\Sigma }}_{i,j}=(I-K_{i,j}^{*}C^{*})P_{i,j-1}$
17.	→ Where * denotes the missing data modifications proposed by Shumway & Stoffer [11]
18.	→ Correction step using the current survival data: $p(x_{i,j}|y_{i,1\;:\;j}^{\left(O\right)},t_{i,j+1},{\bar{\delta }}_{i,1\;:\;j})\approx N({\tilde{\mu }}_{i,j},{\tilde{\Sigma }}_{i,j})$
19.	→ Find ${\tilde{\mu }}_{i,j}=\arg\min_{x_{i,j}}g(x_{i,j})$ and ${\tilde{\Sigma }}_{i,j}={\left(H_{g}{|}_{x_{i,j}={\tilde{\mu }}_{i,j}}\right)}^{-1}$ where *H*_*g*_ is the Hessian matrix of $g(x_{i,j})$
20.	→ Where $g(x_{i,j})=-{\bar{\delta }}_{i,j}\gamma^{⊤}\omega_{i}-{\bar{\delta }}_{i,j}\alpha^{⊤}Hx_{i,j}+\tau_{i,j}\exp\left\{\gamma^{⊤}\omega_{i}\right\}\exp\left\{\alpha^{⊤}Hx_{i,j}\right\}+\frac{1}{2}{\left(x_{i,j}-{\overset{ˇ}{\mu }}_{i,j}\right)}^{⊤}{\overset{ˇ}{\Sigma }}_{i,j}^{-1}(x_{i,j}-{\overset{ˇ}{\mu }}_{i,j})$
21.	→ For every time step *j* = *m*_*i*_ − 1, …, 1 (Smoothing):
22.	→ $p(x_{i,j}|y_{i,1\;:\;m_{i}},T_{i},{\bar{\delta }}_{i,1\;:\;m_{i}})\approx N({\hat{\mu }}_{i,j},{\hat{\Sigma }}_{i,j})$
23.	→ ${\hat{\mu }}_{i,j}={\tilde{\mu }}_{i,j}+J_{i,j}({\hat{\mu }}_{i,j+1}-A{\tilde{\mu }}_{i,j})$
24.	→ ${\hat{\Sigma }}_{i,j}={\tilde{\Sigma }}_{i,j}+J_{i,j}({\hat{\Sigma }}_{i,j+1}-A{\tilde{\Sigma }}_{i,j}A^{⊤}-W)J_{i,j}^{⊤}$
25.	→ $J_{i,j}={\tilde{\Sigma }}_{i,j}A^{⊤}{\left(A{\tilde{\Sigma }}_{i,j}A^{⊤}+W\right)}^{-1}$
26.	→ Where ${\hat{\mu }}_{im_{i}}={\tilde{\mu }}_{im_{i}},\;{\hat{\Sigma }}_{im_{i}}={\tilde{\Sigma }}_{im_{i}}$
27.	→ Evaluate the required expectations using equations (3.13)–(3.21)
28.	→ **M Step**:
29.	→ Use equations (3.24)–(3.30) to update the parameters $\theta^{\left(k\right)}$

## Appendix D. Histograms for estimated parameter values within the simulation study

See figure 7.

## Appendix E. Survival predictions for biased parameter estimates

In the scenario with 75% missing observations within the simulation study, the survival parameters exhibited bias. To further scrutinize this bias, figure 8 displays a scatter plot comparing predicted survival values from the estimated model against those derived from the true model on a testing dataset in a simulation where parameter bias was evident $\left(\gamma ={\left[1.9384\;-0.7275\right]}^{⊤},\alpha =-1.1214\right)$. This plot illustrates that across the entire range of predicted survival values, both models yield comparable results. Minor deviations, such as slight overestimation and underestimation, may be discerned in the regions 0.2–0.4 and 0.7–0.9, respectively. This suggests that despite the limited number of observations, the estimated model captures the underlying trend of interest, albeit with parameter values differing from those of the true model. Thus, it appears that with the available measurements, multiple solutions exist that produce similar survival curves to those obtained through the true model.

## Appendix F. Area under the receiver operating characteristic curve in simulations

Figure 9 presents two plots depicting the average area under the ROC curve (AUC) for two landmarks across various horizons in the 50% missing measurements setup across all runs. Other simulation settings show similar results. It is noticeable that the model’s discriminatory capability diminishes with longer horizons, suggesting that as forecasts extend further into the future, the model struggles slightly more to differentiate those who experience the event. This phenomenon could stem from the Markovian assumption and the increased uncertainty in hidden state distributions, ultimately resulting in slightly less precise survival predictions for further time points in the future.

## Appendix G. Examples of patient longitudinal trajectories

Two patients from the PAH data are depicted in figure 10, showcasing their leading hidden state trajectories alongside observed walking distance measurements. It is evident that the expected values for the hidden states effectively model the longitudinal observations and are accurately tracked. Consequently, the model successfully identifies individualized trajectories based on the available data for each patient.
